# The Practice of Surgery: Embracing Minor Surgery and the Application of Dressings, Etc.

**Published:** 1850-02

**Authors:** 


ART. IV.—*The Practice of Surgery: embracing Minor Surgery and the application of dressings, etc.* By JOHN HASTINGS, M. D., U. S. N. Fellow of the College of Physicians, of Philadelphia; member of the Philadelphia Medical Society; Lecturer on Surgical Anatomy and operative Surgery, etc. With numerous illustrations. Philadelphia: Lindsay & Blakiston, 1850.

The enterprising publishers, Messrs. Lindsay & Blackiston, could not have treated the profession with a more charming New Year's gift. The volume is neatly got up, with good paper and clear type, and *its author is a Yankee!* i. e. we mean to say he is a "home-bred" gentleman, and the book is, therefore, of "home" manufacture. We have found the matter well arranged and orthodox.

If it be justly said of us that we have written but few surgical books, it is at least flattering to our self pride to know that whatever we have written has been respectable. Dorsey, Gibson, Anderson, Burke, Parrish, Warren, N. R. Smith, McLellan, Pancoast, Mutter, Larquit, with others, have given us a surgical literature of our own, which, in point of sound practical matter and method, will well compare with that of any other country. We sincerely wish that American surgeons would more often read American books, and they would learn to appreciate better our native experience and talent. F. H. H.

For sale by Derby & Co., Buffalo.

